# Current Applications of Genetic Risk Scores to Cardiovascular Outcomes and Subclinical Phenotypes

**DOI:** 10.1007/s40471-015-0046-4

**Published:** 2015-07-01

**Authors:** Jennifer A. Smith, Erin B. Ware, Pooja Middha, Lisa Beacher, Sharon L. R. Kardia

**Affiliations:** Department of Epidemiology, School of Public Health, University of Michigan, 1415 Washington Heights, Ann Arbor, MI 48109 USA; Research Center for Group Dynamics, Institute for Social Research, University of Michigan, Ann Arbor, MI 48104 USA

**Keywords:** Genetic risk score, Cardiovascular disease, Coronary heart disease, Ischemic stroke, Hypertension, Blood pressure

## Abstract

Genetic risk scores are a useful tool for examining the cumulative predictive ability of genetic variation on cardiovascular disease. Important considerations for creating genetic risk scores include the choice of genetic variants, weighting, and comparability across ethnicities. Genetic risk scores that use information from genome-wide meta-analyses can successfully predict cardiovascular outcomes and subclinical phenotypes, yet there is limited clinical utility of these scores beyond traditional cardiovascular risk factors in many populations. Novel uses of genetic risk scores include evaluating the genetic contribution of specific intermediate traits or risk factors to cardiovascular disease, risk prediction in high-risk populations, gene-by-environment interaction studies, and Mendelian randomization studies. Though questions remain about the ultimate clinical utility of the genetic risk score, further investigation in high-risk populations and new ways to combine genetic risk scores with traditional risk factors may prove to be fruitful.

## Introduction

Multi-cohort genome-wide association studies (GWAS) have now identified hundreds of genetic variants that are credibly associated with cardiovascular outcomes, subclinical cardiovascular phenotypes, and risk factors for cardiovascular disease (CVD). However, the individual genetic variants, or single nucleotide polymorphisms (SNPs), that have been identified typically explain a very small fraction of the variation in complex traits and thus have limited predictive capacity for disease risk [1]. Aggregating information about multiple SNPs, each with small effects, into a single genetic risk score (GRS) has become a useful tool for examining the cumulative predictive ability of genetic variation at known loci on cardiovascular disease outcomes and related phenotypes [2].

Here, we outline key aspects of creating GRSs, discuss their quantification and evaluation, and provide a brief summary of the predictive ability of GRSs for cardiovascular outcomes and subclinical CVD phenotypes. Emerging uses of GRSs will then be discussed, including (1) prediction in clinical and high-risk populations, (2) GRS-by-environment interaction studies, and (3) Mendelian randomization studies. Cardiovascular outcomes discussed include coronary heart/artery disease (CHD/CAD), myocardial infarction (MI), ischemic stroke (IS), hypertension (HTN), and a composite CVD phenotype that includes both heart disease and stroke (also conceptualized as “total cardiovascular diseases” [3]). Subclinical phenotypes include artery calcification and intimal-medial thickness (IMT).

## Creating Genetic Risk Scores

Fundamentally, the creation of a GRS involves summarizing information across multiple SNPs. The most common method sums the number of risk-conferring alleles that an individual has (0, 1, or 2) across all loci. A statistically analogous coding scheme is to assign the heterozygous state (i.e., Aa) a value of 0, the non-risk homozygous state −1, and the risk homozygous state 1. If an individual is missing a small proportion of genotype data needed to construct the GRS (such as 1 or 2 SNPs), imputation to the most common genotype category is commonly used. An alternative to imputation is to only include SNPs that have complete genotype data in the GRS, followed by GRS rescaling to be consistent with GRSs created using all SNPs.

### SNP Selection

To create a GRS, one must first select the genetic variants to be included in the risk score. Although earlier studies creating GRSs for cardiovascular traits included SNPs from biologically plausible candidate gene association studies [4, 5], most current GRSs are constructed using SNPs found to be associated with traits through GWAS. The standard in the field has been to use SNPs that reached genome-wide significance (*p* < 5 × 10^−8^) in large, consortium-based, multi-study GWAS meta-analyses. Typically, these consortium-based meta-analyses also include a replication phase, which further enhances the robustness of the findings. While selecting SNPs from large meta-analyses is considered the gold standard, less preferable strategies for SNP selection may be used if meta-analyses have not yet been conducted for the trait of interest in populations that are demographically and/or ethnically similar to the population under study (for example, SNPs may be selected from biologically plausible candidate genes or from a single-study GWAS). The vast majority of GRSs in the literature include only common variants (SNPs with minor allele frequency (MAF) > 5 %), because meta-analyses tend not to have sufficient power to detect effects from rarer variants.

A disadvantage of including only the most highly significant and replicated SNPs is that there may be many other SNPs with true effects that do not reach the stringent genome-wide significance levels. Recent work evaluating the relationship between coronary artery calcification (CAC) and GRSs constructed from CHD/MI-associated SNPs at various meta-analysis *p* value thresholds showed that trait variation for CAC is maximally explained by including thousands of SNPs that are at least marginally associated with CAD/MI (*p* < 0.2) in the GRS [6•]. On the other hand, research on type 2 diabetes suggests that GRSs constructed from increasing numbers of SNPs may not substantively improve risk prediction [7]. With these limited, sometimes conflicting results, more work is needed to verify whether inclusion of additional marginally significant SNPs in GRSs is the best approach for cardiovascular traits.

It has also been suggested that relying solely on genome-wide significant SNPs from the largest, most current meta-analysis may not be the best approach to SNP selection. New algorithms that integrate information from multiple sources into GRS SNP selection are beginning to appear. For example, Belsky and colleagues implemented a novel SNP selection method using public-access resources, including GWAS results databases and web-based GWAS analysis tools, to select SNPs from 16 published GWAS for an obesity GRS [8••]. This type of algorithm may represent a systematic and replicable method for integrating results from a wider variety of sources.

### Weights

When equal weights are assigned to each genetic variant, the score is “unweighted,” and its construction is based on the assumption that each risk allele confers identical risk. However, for most complex traits, effect sizes across identified SNPs vary (see, for example, [9]). Thus, GRSs are often constructed by weighting SNPs by their GWAS meta-analysis effect sizes, thus giving more weight to variants with stronger effects. Weighted scores may increase statistical power compared to unweighted scores, provided that the weights are accurately determined [10••]. Weights are ideally calculated from consortium-based meta-analysis effect sizes, which are more precise due to large sample sizes. This weighting method is commonly used when the target population (the population in which the GRS is going to be evaluated) has a similar demographic and ethnic composition as the meta-analysis population (the population used to estimate the effect sizes). See below for a more detailed discussion of the importance of ethnicity in GRS creation. An unweighted score is often the best option if there are no stable effect estimates available because (1) no GWAS meta-analyses have yet been performed on the trait of interest (and thus SNPs are selected from candidate gene studies or small, un-replicated GWAS), (2) existing meta-analyses are comprised of studies with different ethnicities or demographic profiles than the population to be studied, or (3) SNPs identified using multiple traits on different measurement scales are to be combined into a single GRS (for example, a GRS that comprises SNPs associated with multiple “intermediate traits,” described in greater detail below).

### Population-Specific Considerations

A potential disadvantage of selecting SNPs based on published meta-analysis results is that many meta-analyses for complex traits have been conducted solely in European-ancestry (EA) populations. This practice is problematic because the SNPs most significantly associated with a trait often differ across ethnicities for a variety of reasons including (1) ethnicity-specific genetic variation, (2) allele frequency differences across ethnicities, and (3) differing patterns of linkage disequilibrium (LD) resulting in ethnicity-specific “tag SNPs” that are associated with the causal variant(s) [11]. Trans-ethnic meta-analyses for cardiovascular traits are beginning to emerge [12, 13] and may offer several advantages over single-ethnicity analyses [11]. However, until trans-ethnic analyses become commonplace, other approaches for GRS SNP selection may be required for GRSs constructed for use in non-EA populations. One strategy is to evaluate SNPs from EA meta-analyses for association within the target population and retain only those that have at least a marginal effect. SNPs selected in this manner may also be combined with the most highly significant SNPs from smaller ethnicity-specific GWAS meta-analyses (see, for example, [14, 15]).

Population-specific factors such as age, sex, and demographics may also be important considerations for SNP selection and weighting. While there is an awareness that confounding and effect modification by population-specific factors may influence both estimates and inferences, the field of genetics has been primarily concerned with race/ethnicity because of the way in which it fundamentally changes the variants that are included and identified in an analysis. Aside from ethnicity, GWASs often include populations with a large range of demographics in order to achieve the sample sizes necessary to obtain enough power to accurately identify SNPs. Nevertheless, glaring demographic differences should be considered when creating GRSs.

## Estimating and Evaluating Genetic Risk Score Effects

The effect sizes for the associations between GRSs and cardiovascular phenotypes are typically reported as beta estimates, odds ratios (ORs), or hazard ratios (HRs), as appropriate for the type of outcome. Effects may be reported per risk allele (corresponding to a one-allele increase in GRS), per GRS standard deviation (SD), or with respect to a particular comparison such as the contrast between the highest and lowest GRS quartiles. Differing methods of reporting effects often makes comparison across GRS studies difficult. In addition, per allele effect sizes tend to decrease as newly discovered variants are incorporated into GRSs. This is due to smaller effect sizes of the newly discovered variants compared to those discovered in the first wave of GWAS meta-analysis, as has been demonstrated for type 2 diabetes [9]. Thus, reporting effects per GRS SD is more effective for cross-study and cross-trait comparisons.

The contribution of GRSs to quantitative cardiovascular traits, such as CAC, is typically reported as the percent of variation in the trait explained by the GRS. This may be assessed before and after adjustment for traditional cardiovascular risk factors (e.g., body mass index (BMI), lipids, HTN, diabetes, and others). For clinical outcomes, such as CAD or HTN, the predictive capacity of the GRS is most commonly evaluated using metrics for risk discrimination and risk reclassification (reviewed in [16]). Prediction models are constructed before and after including traditional cardiovascular risk factors and/or family history of disease. If the GRS significantly improves prediction after inclusion of traditional risk factors, it demonstrates the potential for clinical utility through more accurate disease risk prediction for patient populations. The area under the receiver operating curve (AUC, or c-statistic) is commonly used to assess discrimination between people with and without disease [17–19]. The c-index is the analogous measure for survival data. Higher AUCs indicate more accurate discrimination, and model improvement is assessed by change in AUC across models. However, many have argued that AUC-based methods may not be optimal for predicting risk [19]. The net reclassification improvement index (NRI) is a popular choice for evaluating risk reclassification [16, 20, 21, 22••]. This statistic evaluates a prediction model’s ability to correctly reassign individuals into disease classifications when compared to a different model. Positive values of NRI correspond to prediction improvement. Clinical NRI corresponds to correct risk reclassification of individuals at intermediate risk for disease and may be more clinically relevant than the traditional NRI [23]. Other methods for assessing the potential for clinical utility, such as the integrated discrimination improvement (IDI) [20], are also available.

## Applying GRSs to Cardiovascular Traits

A variety of approaches have been used to examine the relationship between GRSs and cardiovascular traits. Below, we discuss the current literature on (1) CAD/CHD-associated SNPs predicting CAD/CHD, (2) blood pressure (BP)-associated SNPs predicting BP and HTN, both within and across ethnic groups, (3) intermediate trait-GRSs predicting cardiovascular outcomes, (4) GRSs predicting composite CVD, and (5) GRSs associated with subclinical measures of heart disease. Representative examples of studies that fall into each of these categories are provided for dichotomous cardiovascular outcomes and quantitative cardiovascular traits in Tables 1 and 2, respectively.

**Table 1:** Examples of studies evaluating the relationship between genetic risk scores and cardiovascular outcomes

Reference (first author, year)^a^	Outcome^b^	Sample^c^	Source of SNPs for GRS^d^	GRS (no. of SNPs, weights)^e^	Effect estimate^f^	Clinical utility^g^
Paynter, 2010 [39]	I-CVD	19,313 EA (women)	CVD outcomes (multiple sources from [57])	12, U	HR^h^ = 1.04 (1.00–1.08) per risk allele	Δ*C* index = 0.001 (*p* = 0.12) NRI = 0.05 (*p* = 0.59)
Cox et al., 2014 [41]	CVD, CVD mortality	1,175 EA (T2D cases)	CHD/MI (7 sources, see [40])	13, W	CVD^i^: OR = 1.51 (1.22–1.86) per risk allele CVD mortality^i^: HR = 1.35 (1.01–1.81) per risk allele	CVD: NRI = 0.03 (*p* = 0.13) CVD mortality: NRI = 0.06 (*p* = 0.06)
Tikkanen, 2013 [24]	I-CHD, I-CVD	24,124 N. Eur	MI, CHD (4 sources, see [24])	28, W	I-CHD^j^: HR = 1.27 (1.20–1.35) per GRS SD I-CVD^j^: HR = 1.18 (1.12–1.24) per GRS SD	For I-CHD, Δ*C* index ≈ 0.004 (***p*** **≤ 0.001**) NRI for CHD = 0.05 (***p*** = **0.01**) Clinical NRI for CHD = 0.27 (***p*** **= 1.1** × **10** ^–**8**^)
Vaarhorst, 2012 [36]	I-CHD	2,559 EA	CHD (multiple sources from [57])	29, W	HR^k^ = 1.12 (1.04–1.21) per risk allele	Δ*C* index = 0.002 (***p*** = **0.13**) NRI = 0.03 (***p*** = **0.03**)
Havulinna, 2013 [28]	I-CHD, I-stroke, I-CVD	32,669 N. Eur	SBP, DPB (4 sources, see [28])	32, W	For GRS quantiles 5 vs. 1, I-CHD^l^, GRS_SBP_: HR = 1.25 (1.07–1.46) I-CHD^l^, GRS_DBP_: HR = 1.23 (1.05–1.43) I-Stroke^l^, GRS_SBP_: HR = 1.24 (1.01–1.53) I-Stroke^l^, GRS_DBP_: HR = 1.35 (1.09–1.66) I-CVD^l^, GRS_SBP_: HR = 1.23 (1.08–1.40) I-CVD^l^, GRS_DBP_: HR = 1.26 (1.11–1.44)	For 10-year I-CVD using GRS_SBP_, NRI = −0.0008 (*p* = 0.92) Clinical NRI = 0.04 (***p*** = **5 × 10** ^−**5**^)
Fava, 2014 [31]	IS	6,092 N. Eur	SBP, DPB (4 sources, see [31])	29, W	OR^m^ = 1.09 (1.03–1.15) per GRS SD	ΔAUC = 0.003 (*p* > 0.05) NRI = 0.07 (***p*** = **0.013**)
Ibrahim-Verbaas, 2014 [35•]	IS, all stroke	22,720 EA (age > 55 years)	Stroke, 9 risk factors (>30 sources, see [35•])	324, W	–	IS^n^: ΔAUC = 0.02 (***p*** = **3.7** × **10** ^−**7**^) All stroke^n^: ΔAUC = 0.02 (***p*** = **2.3** × **10** ^−**6**^)
Ehret, 2011 [26]	HTN	23,294 EA (women)	SBP, DBP [26]	29, W	HTN^o^: OR = 1.23 (1.19–1.28) per GRS SD	–

*p* values < 0.05 are in bold

*I* incident, *EA* European ancestry, *N. Eur.* Northern European ancestry, *W* weighted, *U* unweighted, *SD* standard deviation, Δ change, *SNP* single nucleotide polymorphism, *GRS* genetic risk score, *GWAS* genome-wide association study, *CI* confidence interval, *CVD* cardiovascular disease, *CHD* coronary heart disease, *IS* ischemic stroke, *HTN* hypertension, *MI* myocardial infarction, *SBP* systolic blood pressure, *DBP* diastolic blood pressure, *HDL*-*C* high-density lipoprotein cholesterol, *T2D* type 2 diabetes, *BMI* body mass index, *HR* hazard ratio, *OR* odds ratio, *NRI* net reclassification improvement index, *AUC* area under the receiver-operator curve

^a^Only selected GRS analyses from each study are reported in this table

^b^Cardiovascular outcome tested for association with GRS

^c^Refers only to the sample used for evaluating the association between the outcome and GRS, corresponding to the effect estimates listed in this table

^d^All studies used GWAS/meta-analyses as the SNP source for the GRS. The outcome/trait used in the GWAS is reported

^e^Number of SNPs in the GRS

^f^Effect estimate (95 % CI) for association between outcome and GRS. Adjustment variables are listed below

^g^Estimates for clinical utility compare a prediction model including the GRS and traditional CVD risk factors (i.e., the adjustment variables described in the effect estimate column) to a model with only traditional CVD risk factors, followed by *p* value

^h^Adjustment variables: Age, SBP, hypertensive medication use, smoking, diabetes, total cholesterol, HDL-C

^i^Adjustment variables: Age, sex, T2D, BMI, current smoking, HTN, dyslipidemia

^j^Adjustment variables: Sex, total cholesterol, HDL-C, BMI, SBP, antihypertensive treatment, smoking, T2D

^k^Adjustment variables: Sex, current smoking, SBP, total cholesterol, HDL-C, self-reported diabetes, BMI, parental history of MI

^l^Adjustment variables: Age, age squared, sex

^m^Adjustment variables: Age, sex, T2D, smoking habits, HTN

^n^Adjustment variables: Age, sex, Framingham Stroke Risk Score (SBP, T2D, smoking, prior CVD, atrial fibrillation, left ventricular hypertrophy, antihypertensive medication)

^o^Adjustment variables: Age, age-squared, BMI

**Table 2:** Examples of studies evaluating the relationship between genetic risk scores and quantitative cardiovascular traits

Reference (first author, year)^a^	Trait^b^	Sample^c^	Source of SNPs for GRS^d^	GRS (no. of SNPs, weights)^e^	Effect estimate^f^
Ehret, 2011 [26]	SBP, DBP	23,294 EA (women)	SBP, DBP [26]	29, W	SBP^g^: *β* = 1.65 (0.10) mmHg per GRS SD (***p*** **= 6.5** × **10** ^−**63**^) DBP^g^: *β* = 1.06 (0.07) mmHg per GRS SD (***p*** **= 8.4** × **10** ^−**57**^)
Fava, 2013 [27]	SBP, DBP, ΔSBP, ΔDBP	Up to 16,375 N. Eur	SBP, DBP (3 sources, see [27])	29, U	SBP^h^: *β* = 0.97 (0.10) mmHg per GRS SD (***p*** **= 2.8** × **10** ^−**21**^) DBP^h^: *β* = 0.59 (0.06) mmHg per GRS SD (***p*** **< 6.7** × **10** ^−**20**^) ΔSBP^h^: *β* = 0.03 (0.01) mmHg/year per GRS SD (***p*** **= 3.3** × **10** ^−**05**^) ΔDBP^h^: *β* = 0.02 (0.004) mmHg/year per GRS SD (***p*** **= 3.5** × **10** ^−**07**^)
Lu, 2015 [14]	SBP, DBP	28,048 Asian	SBP, DBP (multiple sources including [14], see [14])	19, W	SBP^i^: *p* = **4.73** × **10** ^−**67**^ for slope of mean SBP across GRS groups DBP^i^: ***p*** **= 2.03** × **10** ^−**69**^ for slope of mean DBP across GRS groups
Bos, 2013 [42]	Artery calcification	1,987 EA	CAC [58]	3, W	For ln [calcification volume +1.0 mm^3^] per GRS SD increase, Aortic arch calcification: 0.15 (0.11–0.20)^j^, 0.01 (−0.03 to 0.06)^k^ Extracranial carotid calcification: 0.14 (0.10–0.18)^j^, 0.04 (0.00–0.08)^k^ Intracranial carotid calcification: 0.11 (0.07–0.16)^j^, 0.00 (−0.04 to 0.04)^k^
Isaacs, 2013 [43]	Carotid plaque score	10,399 EA	TC, LDL-C, HDL-C, TG [59]	52, W (GRS_TC_) 37, W (GRS_LDL-C_) 47, W (GRS_HDL-C_) 32, W (GRS_TG_)	Carotid plaque^l^: *β* = 0.11 (0.02) per GRS_TC_ SD (***p*** **= 4.91** × **10** ^−**8**^) Carotid plaque^l^: *β* = 0.11 (0.02) per GRS_LDL-C_ SD (***p*** **= 3.18** × **10** ^−**8**^) Carotid plaque^l^: *β* = 0.05 (0.02) per GRS_HDL-C_ SD (***p*** **= 0.01**) Carotid plaque^l^: *β* = 0.05 (0.02) per GRS_TG_ SD (***p*** **= 0.01**)
van Setten, 2015 [6]	CAC	2,599 EA	CAD/MI [12] LDL [60] TC [60] BMI [61] T2D [62]	8,918, W (GRS_CAD/MI_) 58, W (GRS_LDL-C_) 72, W (GRS_TC_) 31, W (GRS_BMI_) 55, W (GRS_T2D_)	PVE^m^ = 1.50 % for GRS_CAD/MI_ (***p*** **= 1.6** × **10** ^−**11**^) PVE^n^ = 0.25 % for GRS_LDL-C_ (***p*** **= 1.6** × **10** ^−**3**^) PVE^n^ = 0.15 % for GRS_TC_ (***p*** **= 8.5** × **10** ^−**3**^) PVE^n^ = 0.25 % for GRS_BMI_ (***p*** **= 2.6** × **10** ^−**2**^) PVE^n^ = 0.25 % for GRS_T2D_ (***p*** **= 4.0 × 10** ^−**2**^)

*SNP* single nucleotide polymorphism, *GRS* genetic risk score, *GWAS* genome-wide association study, *CI* confidence interval, *SBP* systolic blood pressure, *DBP* diastolic blood pressure, *BP* blood pressure, *CAC* coronary artery calcification, *CAD* coronary artery disease, *MI* myocardial infarction, *TC* total cholesterol, *LDL*-*C* low-density lipoprotein cholesterol, *HDL*-*C* high-density lipoprotein cholesterol, *TG* triglycerides, *BMI* body mass index, *T2D* type 2 diabetes, *EA* European ancestry, *N. Eur.* Northern European ancestry, *AA* African ancestry, *Asian* Asian ancestry, *W* weighted, *U* unweighted

^a^Only selected GRS analyses from each study are reported in this table

^b^Cardiovascular trait tested for association with GRS; Δ denotes change over time

^c^Refers only to the sample used for evaluating the association between the trait and GRS, corresponding to the effect estimates listed in this table

^d^All studies used GWAS/meta-analyses as the SNP source for the GRS. The outcome/trait used in the GWAS is reported

^e^Number of SNPs in the GRS

^f^Effect estimate (standard error, 95 % CI) or percent variance explained (PVE) for association between trait and GRS (SD = standard deviation). *P* values are listed in parentheses, and *p* values < 0.05 are in bold. When effect estimates are not available, only *p* values are provided. Adjustment variables are listed below

^g^Adjustment variables: Age, age-squared, BMI (adjustment variables)

^h^Adjustment variables: Basic demographic, anthropometric, anamnestic, socioeconomic, lifestyle data, gluco-lipid parameters, estimated glomerular filtration rate, follow-up years (when appropriate)

^i^Adjustment model not specified. GRS groups were defined by one risk allele increments for SBP analysis and 0.5 risk allele increments for DBP analysis; slope of mean SBP or DBP was estimated across GRS groups

^j^Unadjusted

^k^Adjustment variables: Age, sex, BMI, SBP, DBP, BP-lowering medication, T2D, TC, lipid-lowering medication, smoking status

^l^Adjustment variables: Age, sex, current and former smoking, HTN, BMI, T2D, alcohol consumption

^m^Adjustment variables: Age, smoking, first genetic principal component, three known CAC risk SNPs (see [6•]), 45 known CAD/MI risk SNPs from [12]

^n^Adjustment variables: Age, smoking, first genetic principal component

### CAD/CHD-Associated SNPs Predicting CAD/CHD

The original concept of using GRSs to predict cardiovascular disease focused on using SNPs associated with the trait of interest to predict that same trait (i.e., CAD-GRS to predict CAD). Several studies have used SNPs found to be associated with CAD in consortium-based meta-analyses to construct GRSs for evaluation with respect to CAD/CHD in EA populations. For example, a weighted 24-SNP GRS constructed using CAD-associated SNPs from four meta-analyses was significantly associated with incident CHD in a Finnish cohort of >24,000 individuals (HR = 1.27 per GRS SD) [24]. A study of >10,000 Swedes showed similar results for a 46-SNP GRS constructed using CAD-associated SNPs from the largest CAD meta-analysis to date [12] (HR = 1.54 for incident CHD comparing first versus fourth GRS quartile) [25]. In both studies, as is often the case when using GRSs to predict cardiovascular disease outcomes, models including the GRS modestly but significantly improved risk reclassification beyond traditional risk factors, but discrimination was not improved.

### BP-Associated SNPs Predicting BP and HTN

The strength of association between GRSs constructed from BP-associated SNPs and HTN or BP has been examined in EA populations. In 2009, the International Consortium for Blood Pressure Genome-Wide Association Studies consortium (ICBP) conducted a GWAS meta-analysis of HTN and BP phenotypes in >200,000 EA [26]. A GRS was created from 29 SNPs associated with systolic blood pressure (SBP) and/or diastolic blood pressure (DBP) at *p* < 5 × 10^−9^, weighted by the mean effect size for SBP and DBP. The GRS was evaluated in an independent cohort of 23,294 women and showed an increase of 1.65 and 1.10 mmHg per SD of the GRS for SBP and DBP, respectively, as well as a 23 % increase in the odds of HTN. When this same GRS was evaluated in a longitudinal study of >17,000 Swedes, a 1 SD increase in GRS was significantly associated with an increase of 1.0 and 0.6 mmHg in SBP and DBP, respectively, as well as a 61 % increase in the odds of hypertension at baseline [27]. The proportion of variation explained by the GRS was 1.0, 0.7, and 2.9 % for SBP, DBP, and HTN, beyond traditional risk factors. Changes in SBP (beta = 0.03 mmHg), DBP (beta = 0.023 mmHg), and HTN incidence (OR = 1.11) were also significantly associated with the GRS. In this study, discrimination for HTN was marginally but not significantly improved by adding the GRS to traditional risk factors. These studies show that while GRSs created for BP and HTN are strongly associated with these traits, clinical utility may be limited.

GRSs consisting of BP-associated SNPs have also been evaluated in non-EA ethnicities. A trans-ethnic GWAS meta-analysis with an African-ancestry discovery sample and a multi-ethnic replication sample identified five SNPs credibly associated with blood pressure that were not previously identified through EA meta-analyses [13]. In the African-ancestry discovery sample, a weighted GRS with these five SNPs explained 0.44 and 0.54 % of the variation in SBP and DBP, respectively, after adjustment for age, body mass index, gender, and the top ten genetic principal components (to account for ancestry). A composite score that included the five SNPs along with the 29 ICBP variants [26] explained 0.80 and 1.42 % of the variation in SBP and DBP. This illustrates that GRSs constructed from SNPs identified in EA-only meta-analyses are often associated with the same traits in other ethnicities but to a lesser degree than in EA samples. In addition, ethnicity-specific SNP identification often leads to an increase in predictive capacity for GRSs, underscoring the need for GWAS meta-analyses that include multiple ethnic groups.

A GWAS meta-analysis with over 80,000 Han Chinese (including discovery and replication samples) identified several SNPs that met genome-wide significance for association with SBP, DBP, and/or HTN [14]. A GRS was constructed that included these SNPs as well as SNPs from previous GWAS meta-analyses that were conducted in EA-only or East Asian-only samples. Prior to inclusion in the GRS, SNPs identified in the EA or East Asian meta-analyses were screened to have at least nominally significant associations with BP in the Han Chinese sample. In a subset of >28,000 subjects, the GRS was significantly associated with HTN (OR = 1.66 for the highest versus lowest quintile of GRS) and was also significantly associated with SBP and DBP. This study illustrates a well-powered hybrid approach to GRS SNP selection for use in non-EA ethnic groups.

### Intermediate Trait-GRSs Predicting Cardiovascular Outcomes

In order to gain a better understanding of the relative genetic contribution of specific intermediate pathways or risk factors to cardiovascular traits, GRSs constructed from SNPs associated with intermediate traits (e.g., CAC) may be evaluated for association with cardiovascular outcomes such as CHD, stroke, and CVD. This approach can augment or extend findings from studies using GRSs constructed from trait-specific SNPs. For example, GRSs constructed from BP-associated SNPs (SBP and DBP separately) were associated with incident CHD, IS, and CVD in a large cohort of Finnish subjects (HRs for CHD = 1.25 and 1.23; HRs for IS = 1.25 and 1.35; HRs for composite CVD = 1.23 and 1.26 for GRS_SBP_ and GRS_DBP_, respectively). This study illustrates that the genetic factors that influence BP also have a significant effect on clinical outcomes [28].

In some cases, constructing GRS based on intermediate traits may be the only option due to the relative lack of studies or replicable significant findings from meta-analyses for the trait itself. For example, large-scale meta-analyses of stroke or IS are just beginning to emerge ([29, 30], but the sample sizes are typically smaller for this cardiovascular trait than other CVD outcomes (such as CHD) or for intermediate traits (such as BP or lipids). Several studies have evaluated the relationship between intermediate trait or risk factor-based GRSs and IS. For example, a GRS constructed from SNPs associated with BP was significantly associated with IS in a Swedish sample of 3,677 stroke cases and 2,415 controls (OR = 1.09 per SD increase in GRS) [31]. The addition of the GRS demonstrated weak but significant improvement in risk reclassification. A GRS constructed from atrial fibrillation-associated SNPs was also significantly associated with incident IS in Swedish participants (HR = 1.23 comparing top and bottom quintiles of GRS) and modestly but significantly improved risk discrimination and reclassification [32]. A GRS constructed from high-density lipoprotein cholesterol (HDL-C) SNPs, however, was not associated with incident IS in European Americans [33].

Recent studies have now gone further to combine SNPs associated with multiple intermediate traits into a composite risk score. For instance, Malik et al. combined SNPs credibly associated with atrial fibrillation, CAD, HTN, and SBP into a single 113-SNP GRS and found that it was significantly associated with IS in both clinic-based case-control and population-based samples (OR = 1.06 per GRS SD in case-control sample) [34]. Adding the GRS improved prediction of IS beyond a sex-adjusted model in the case-control sample but not in the population-based sample. Another emerging strategy for creating a composite GRS is to combine intermediate trait-associated SNPs with SNPs identified for the trait itself. In a meta-analysis of four population-based EA cohorts, a 324-SNP GRS comprised of SNPs credibly associated with stroke and nine stroke risk factors improved discrimination and risk reclassification for IS beyond a well-validated risk factor prediction model [35•].

Studies have also evaluated whether GRSs constructed from CHD-associated SNPs only, intermediate trait-associated SNPs only, or the combination of both types of SNPs are predictive of CHD [25, 36]. A case-control study of individuals in the Netherlands found that the best predictor of CHD was a weighted 29-SNP GRS consisting of CHD-associated SNPs only, with an HR = 1.12 per risk allele after adjustment for traditional risk factors [36]. Other GRSs that included intermediate trait SNPs were less strongly associated with CHD and were attenuated after risk factor adjustment. A separate study in Swedes, however, found that the CHD-specific GRS and the GRS constructed from CHD-plus intermediate trait-associated SNPs were similarly associated with CHD (HR = 1.5 for first vs. fourth quartile of GRS) after adjustment for traditional risk factors, and that risk reclassification was modestly but significantly improved beyond traditional risk factors for both GRSs, although the AUC was not [25]. Taken together, these studies suggest that adding SNPs from intermediate traits to a trait-specific GRS may not improve prediction of cardiovascular outcomes.

### GRSs Predicting CVD as a Composite Phenotype

To date, there have been few consortium-based GWASs that use a composite CVD phenotype (including both heart disease and stroke) as the outcome measure, although some are beginning to emerge [37•]. Instead, GWAS meta-analyses have focused on specific CVD endpoints (such as CAD/CHD, MI, or stroke), subclinical CVD phenotypes (such as CAC, IMT, and plaque), and intermediate phenotypes (for a review, see [38]). Following this trend, many GRSs evaluated for their prediction of CVD have been constructed using these trait-specific SNPs from consortium-based GWAS meta-analyses. Studies that specifically evaluate the shared genetic variation between CAD and stroke may lead to a more refined set of SNPs that best predict composite CVD.

In early work on CVD, Paynter et al. constructed an unweighted 12-SNP GRS from published associations with CVD-related endpoints (*p* < 10^−7^ in meta-analysis) and found a significant relationship with incident CVD in EA women (per-allele HR = 1.05), although this association was attenuated upon adjustment for traditional risk factors [39]. A second unweighted 101-SNP GRS also included SNPs associated with intermediate traits (cholesterol, BP, diabetes, etc.), but the effect of this GRS on CVD was weaker (per-allele HR = 1.02). Thanassoulis et al. found a significant association between incident CVD and an unweighted 13-SNP GRS constructed from MI/CHD-associated SNPs, with HR = 1.05 per allele after adjustment for CVD risk factors and parental history of CVD in the Framingham Heart Study [40]. However, an unweighted 102-SNP GRS that included SNPs associated with intermediate traits was not associated with CVD. These studies show that GRSs with CHD/CAD-associated SNPs are more strongly predictive of composite CVD than more comprehensive GRSs that include intermediate trait-associated SNPs. The same 13-SNP GRS used in Thanassoulis was also found to be significantly associated with prior CVD (OR = 1.51) as well as CVD mortality (HR = 1.35) in EAs with diabetes, after adjustment for CVD risk factors [41]. In all studies, the GRS failed to improve discrimination, although it did modestly improve risk reclassification of some CVD cases in the latter two studies.

### GRSs Associated with Subclinical Measures of Heart Disease

Since only a very small number of SNPs have been reliably associated with artery calcification and IMT, studies have primarily focused on the evaluation of GRSs constructed from CAD/MI and/or other risk factor-associated SNPs with subclinical measures of heart disease. In an EA cohort, a GRS created from three SNPs that have been credibly associated with CAC explained 2.4 % of the variation in CAC, and a 45-SNP GRS constructed from CAD-associated SNPs explained an additional 4 % [6]. Another study conducted in EA found that a GRS from the same three CAC-associated SNPs was associated with calcification in multiple vessel beds, but the associations were no longer significant after adjusting for traditional cardiovascular risk factors [42]. A 132-SNP GRS created from SNPs associated with lipids was also associated with vessel bed calcification, though less strongly than the GRS that contained only CAC-associated SNPs. A third study conducted primarily in EAs found a relationship between plaque and GRSs constructed from lipid-associated SNPs but very limited associations between those GRSs and IMT [43]. However, a separate study found that IMT was associated with a GRS that included five fasting glucose-associated SNPs (beta = 0.0048 mm per GRS SD) [44]. Overall, these studies illustrate that SNPs associated with intermediate traits may be useful for explaining variation in subclinical phenotypes but that more work is needed to identify the SNPs most strongly associated with artery calcification and IMT.

## Emerging Uses of GRS

### Prediction in Clinical and High-Risk Populations

Currently, there is interest in exploring whether GRSs are associated with CVD outcomes and subclinical phenotypes in clinical and other high-risk populations. Accurate prediction of CVD events in high-risk populations, such as those with comorbidities, is imperative because patients are treated according to their risk classification. Initial investigations indicate that GRSs are associated with CVD in populations with comorbidities. For example, as discussed previously, GRSs constructed from CAD/CVD-associated SNPs are associated with CVD, CAC, and CVD mortality in EAs with diabetes, even after adjusting for traditional risk factors [41]. There has also been interest in investigating whether GRSs may be useful for secondary prevention, but most studies have indicated that GRSs are not particularly successful at predicting new CVD events in patients with previous CVD. For example, in a study of 5,742 patients with symptomatic vascular disease, a 30-SNP GRS constructed from CAD-associated SNPs was not able to significantly improve 10-year risk prediction of a composite CVD outcome consisting of MI, stroke, and vascular death [45•]. In a separate study of subjects undergoing heart catheterization, GRSs constructed from CAD/MI-associated SNPs were associated with prevalent, but not incident, MI [46]. More work is needed to assess whether the use of GRSs will translate to clinical utility in terms of risk assessment and ultimately differential treatment in high-risk populations.

### GRS-by-Environment Interaction Studies

Cardiovascular disease is likely to be due, in part, to interaction between genetic and non-genetic components [47], including demographic, dietary, behavioral, environmental, and social factors. Studies of common, chronic diseases have recently begun to utilize GRSs as the genetic variation in gene-by-environment interaction studies, since GRSs cumulatively explain more trait variation than individual SNPs. GRS-by-environment interaction studies are emerging in obesity, type 2 diabetes, and lipids research. For instance, GRS-by-age and GRS-by-BMI interactions have been reported for type 2 diabetes [48, 49], and a GRS-by-education interaction was observed for hemoglobin A1c [50]. Several studies have noted GRS-by-diet interactions, including a GRS-by-sugar sweetened beverages interaction for BMI and obesity [51], a GRS-by-macronutrient intake interaction for adiposity traits [52], and a GRS-by-adiposity interaction for triglycerides and HDL-C [53]. This avenue of research is likely to lead to a greater understanding of the etiological factors that underlie the development of complex diseases and thus represents a promising direction for cardiovascular research.

### GRSs as Instrumental Variables in Mendelian Randomization Studies

Mendelian randomization is a method for obtaining an unbiased estimate of the potential causal effect of a risk factor on an outcome of interest using observational data. With this approach, genetic variants are used as an instrumental variable, or a proxy, for the risk factor. GRSs have become a popular choice for instrumental variables because they typically explain more trait variation than single SNPs [10••]. Recent applications of GRSs in Mendelian randomization studies for cardiovascular diseases include the use of a 14-SNP GRS to explore the causal relationship between HDL-C and MI [54] and an 8-SNP GRS to evaluate the relationship between uric acid and multiple cardiometabolic phenotypes [55]. Burgess and Thompson thoroughly review the use of GRSs as instrumental variables in Mendelian randomization studies and provide simulation studies and recommendations for use [10••]. The extension of Mendelian randomization techniques to other data types, such as epigenetic and metabolomic data, may also be a promising area of research for cardiovascular disease and is discussed in [56].

## Conclusions

As we take stock of the findings from GWAS meta-analyses conducted over the past decade, the GRS has been one of the most promising ways to aggregate multiple sets of results into a single genetic predictor for cardiovascular disease. More work is needed to identify the genetic factors associated with subclinical phenotypes and cardiovascular outcomes, especially in non-EA populations, so that GRSs can most effectively capture relevant genetic variation. Questions remain about the ultimate clinical utility of the GRS, but further investigation in high-risk populations and new ways to combine GRSs with traditional risk factors may prove to be fruitful.
